# Does Dynamic Compliance-Guided PEEP Titration Reduce Postoperative Pulmonary Aeration Loss in Laparoscopic Bariatric Surgery? Randomized Controlled Trial

**DOI:** 10.3390/jcm15114018

**Published:** 2026-05-22

**Authors:** Dilara Göçmen, Yasemin Masatlıoğlu, Feyza Özaltun, Ömer Günal, Tümay Umuroğlu

**Affiliations:** 1 Anesthesiology and Reanimation Department, Marmara University, Istanbul 34899, Türkiye; 2 Anesthesiology and Reanimation Clinic, Pendik Training and Research Hospital, Marmara University, Istanbul 34899, Türkiye; 3 General Surgery Department, Faculty of Medicine, Marmara University, Istanbul 34854, Türkiye

**Keywords:** dynamic compliance, individualized PEEP, laparoscopic bariatric surgery, lung ultrasound, postoperative aeration loss, lung-protective ventilation

## Abstract

**Background and Objectives:** Patients with obesity undergoing laparoscopic bariatric surgery face elevated perioperative pulmonary risk due to impaired respiratory mechanics, reduced functional residual capacity, and pneumoperitoneum-induced atelectasis. Intraoperative changes in intra-abdominal pressure and surgical positioning substantially alter respiratory mechanics, yet studies evaluating repeated PEEP titration at multiple intraoperative time points remain limited. This study aimed to determine whether dynamic compliance-guided individualized PEEP titration, applied at three distinct intraoperative stages, reduces postoperative pulmonary aeration loss compared to fixed 8 cmH_2_O PEEP. **Methods:** In this single-center randomized controlled trial with blinded outcome assessment, 70 patients with obesity (BMI ≥ 35 kg/m^2^) undergoing laparoscopic bariatric surgery were randomized 1:1 to the CDYN group (dynamic compliance-guided PEEP titration at T1: post-induction, T2: during pneumoperitoneum, T3: post-deflation; n = 35) or the PEEP8 group (fixed PEEP 8 cmH_2_O; n = 35). The primary outcome was the modified lung ultrasound score (mLUSS), assessed 30 min after PACU arrival by a blinded investigator (ClinicalTrials.gov: NCT06994780). **Results:** Total mLUSS was significantly lower in the CDYN group (2.20 ± 1.16 vs. 5.80 ± 2.14; *p* < 0.001), with significant differences in both hemithoraces. The PaO_2_/FiO_2_ ratio at PACU was significantly higher in the CDYN group (425.11 ± 127.13 vs. 311.65 ± 92.59 mmHg; *p* < 0.001), and the supplemental oxygen requirement was significantly lower (*p* = 0.001). Dynamic compliance was consistently higher throughout surgery (all *p* < 0.001) without differences in airway pressures or hemodynamics. **Conclusions:** Dynamic compliance-guided individualized PEEP titration, applied at three intraoperative stages, significantly reduces early postoperative pulmonary aeration loss and improves oxygenation in patients with obesity undergoing laparoscopic bariatric surgery, without increasing barotrauma risk or hemodynamic instability.

## 1. Introduction

The global rise in the prevalence of obesity has led to a substantial increase in the demand for laparoscopic bariatric surgery. However, this patient population presents unique perioperative challenges, as an elevated body mass index and increased intra-abdominal pressure exert significant adverse effects on pulmonary mechanics. Increased airway resistance and reduced respiratory compliance heighten the work of breathing in patients with obesity, whereas functional residual capacity (FRC) and respiratory system compliance are markedly diminished. During laparoscopic procedures, pneumoperitoneum causes cephalad displacement of the diaphragm, promoting collapse and atelectasis, predominantly in the dependent lung regions. These mechanical alterations result in increased lung and chest wall elastance and an increase in driving pressure. When combined with a reduced oxygen reserve and compromised respiratory mechanics, this constellation of factors substantially elevates the risk of postoperative pulmonary complications (PPCs) [1].

The appropriate selection of intraoperative ventilation strategies is critical in mitigating these risks. Volume-controlled ventilation (VCV) and pressure-controlled ventilation (PCV) are the most widely employed ventilation modes in laparoscopic bariatric surgery, and positive end-expiratory pressure (PEEP) and recruitment maneuvers are utilized as complementary strategies to preserve pulmonary function throughout the perioperative period [2]. Although various consensus guidelines have emphasized that lung-protective ventilation strategies may reduce the incidence of postoperative pulmonary complications, a universal agreement regarding their optimal clinical implementation has not yet been established [3,4].

The determination of optimal PEEP levels during bariatric surgery remains a subject of ongoing debate. Both low (<10 cmH_2_O) and high (≥10 cmH_2_O) PEEP levels have been investigated; while some studies have suggested that higher PEEP may reduce the incidence of PPCs, others have failed to demonstrate a significant difference between fixed PEEP levels of 8–10 cmH_2_O and zero end-expiratory pressure (ZEEP) [5]. Multicenter randomized controlled trials have reported that the combination of high PEEP and recruitment maneuvers improves intraoperative oxygenation in patients with obesity, yet it confers no significant benefit with respect to PPCs [6]. Furthermore, PEEP requirements among patients with obesity exhibit considerable interindividual variability depending on body mass index, fat distribution pattern, and surgical positioning [7].

While the primary objective of PEEP application is to prevent atelectasis, the risk of alveolar overdistension and barotrauma must be considered equally. Intraoperative conditions, such as Trendelenburg and reverse Trendelenburg positioning, as well as pneumoperitoneum, dynamically alter respiratory compliance and pulmonary resistance throughout the course of surgery. Therefore, rather than applying a fixed PEEP level for the entire procedure, individualized PEEP titration guided by each patient’s real-time respiratory mechanics may offer a more effective lung-protective ventilation strategy [8].

Dynamic compliance (Cdyn) is a parameter that reflects the elasticity of the respiratory system, encompassing both the lungs and chest wall, during the normal breathing cycle and represents the volume change generated per unit change in pressure. In clinical practice, it is calculated by dividing the tidal volume by the difference between the peak inspiratory pressure and PEEP and can be monitored in real time on modern anesthesia workstations. Ventilation management guided by dynamic compliance has been shown to prevent the development of atelectasis and protect lung tissue by minimizing alveolar overdistension (volutrauma) in healthy alveolar units.

Lung ultrasound is a rapid, point-of-care imaging modality that can be readily performed at the bedside without radiation exposure. Although computed tomography remains the gold standard for evaluating pulmonary pathology, its logistical complexity and associated ionizing radiation exposure limit its suitability for the routine diagnosis of perioperative atelectasis. Recent studies have demonstrated that lung ultrasound is an effective and reliable tool for the early detection of pulmonary complications in the operating room and postoperative care unit [9,10,11].

During laparoscopic bariatric surgery, intraoperative changes in intra-abdominal pressure and surgical positioning substantially alter respiratory mechanics, potentially rendering a single pre-incision PEEP titration insufficient to maintain optimal alveolar recruitment throughout the procedure. Despite this physiological rationale, studies evaluating repeated intraoperative PEEP titration at multiple time points—accounting for the sequential effects of pneumoperitoneum and positional changes—remain limited in the literature. The present study addresses this gap by applying dynamic compliance-guided PEEP titration at three distinct intraoperative stages, using the modified lung ultrasound score (mLUSS) as the primary endpoint—a bedside, radiation-free tool that enables objective perioperative assessment of pulmonary aeration without requiring patient transfer or ionizing radiation. Our primary hypothesis was that patients receiving dynamic compliance-guided individualized PEEP titration would demonstrate lower mLUSS values in the early postoperative period compared to those receiving a fixed PEEP of 8 cmH_2_O. Secondary endpoints included the comparison of perioperative oxygenation parameters and the incidence of pulmonary complications within the first four postoperative days between the two groups.

## 2. Materials and Methods

### 2.1. Ethical Consideration

This study was designed as a single-center, prospective, randomized controlled trial with blinded outcome assessment and was conducted at the Asaf Ataseven Supplementary Service Building of Marmara University Faculty of Medicine between April 2025 and January 2026, following approval by the Clinical Research Ethics Committee of Marmara University Faculty of Medicine on 4 March 2025 (No: 09.2025.25.0127; ClinicalTrials.gov: NCT06994780).

### 2.2. Study Population and Patient Selection

A total of 70 patients aged ≥18 years with a body mass index ≥ 35 kg/m^2^, scheduled for laparoscopic bariatric surgery, and who provided written informed consent were enrolled. Patients with severe chronic obstructive pulmonary disease, emphysema, a history of thoracic surgery, severe respiratory failure, or chronic heart failure were excluded.

### 2.3. Study Protocol

Patients were randomized into two groups—PEEP8 and CDYN—by an anesthesiologist using the sealed envelope method in the preoperative assessment room prior to being taken to the operating theater. Following standard anesthetic monitoring, including electrocardiography (ECG), non-invasive blood pressure, bispectral index (BIS™; Medtronic, Dublin, Ireland), and peripheral oxygen saturation (SpO_2_), anesthesia was induced with propofol (1.5–2 mg/kg), remifentanil (0.5–1 mcg/kg), and rocuronium (0.6 mg/kg) in all patients. Invasive arterial monitoring was performed following endotracheal intubation. Mechanical ventilation was delivered using a Mindray Wato EX-65 Pro anesthesia workstation (Mindray Biomedical Electronics, Shenzhen, China) in all patients. All patients were managed intraoperatively by the same anesthesiologist throughout the study period; lung ultrasonography in the postoperative care unit was performed by an experienced anesthesiologist who was blinded to group allocation.

### 2.4. Intraoperative Period

**Figure 1:**
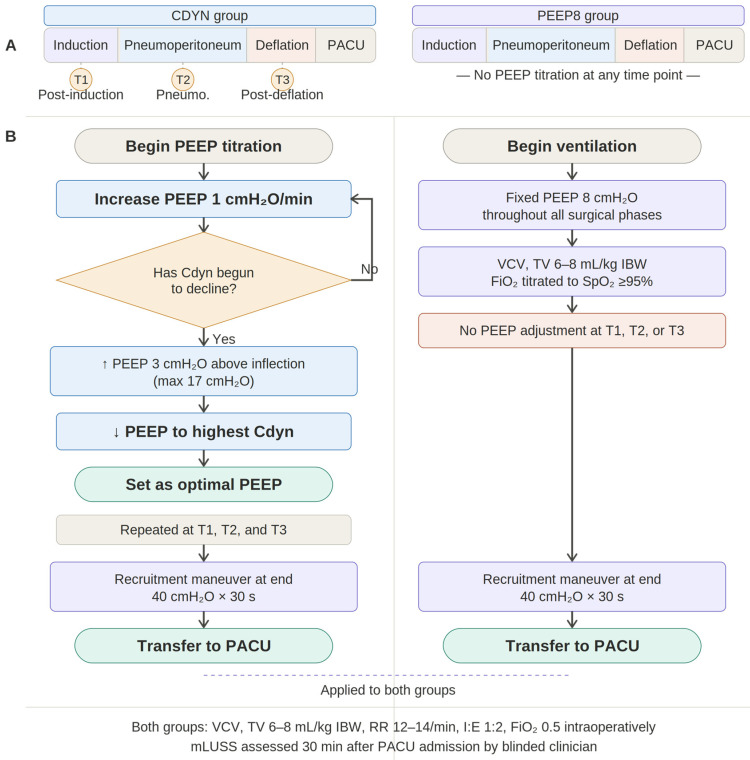
Intraoperative ventilation protocol for the CDYN and PEEP8 groups: (**A**) Surgical timeline showing the three PEEP titration time points in the CDYN group (T1: post-induction; T2: during pneumoperitoneum in reverse Trendelenburg position; T3: following pneumoperitoneum deflation). (**B**) Step-by-step protocol flowcharts for the CDYN group (dynamic compliance-guided individualized PEEP titration) and the PEEP8 group (fixed PEEP 8 cmH_2_O). ↑: incremental increase in PEEP (1 cmH_2_O/min); ↓: decrease PEEP to the level of highest Cdyn. A standardized recruitment maneuver (40 cmH_2_O × 30 s) was applied to all patients in both groups at the end of surgery prior to transfer to the postoperative care unit. CDYN: dynamic compliance-guided PEEP group; PEEP8: fixed 8 cmH_2_O PEEP group; T1–T3: intraoperative PEEP titration time points; mLUSS: modified lung ultrasound score; VCV: volume-controlled ventilation; TV: tidal volume; IBW: ideal body weight.

Patients in Group PEEP8 were ventilated in volume-controlled mode with a tidal volume of 6–8 mL/kg ideal body weight and a fixed PEEP of 8 cmH_2_O. In Group CDYN, PEEP titration was performed at three critical intraoperative time points: following induction (T1), during pneumoperitoneum (T2), and upon pneumoperitoneum deflation (T3). The titration protocol involved incremental increases in PEEP of 1 cmH_2_O per minute, with continuous monitoring of dynamic compliance. Once dynamic compliance began to decline, PEEP was further increased by an additional 3 cmH_2_O beyond that inflection point, up to a maximum of 17 cmH_2_O, and subsequently decreased back to the level at which the highest dynamic compliance value had been recorded. This level was then set as the optimal PEEP for the remainder of that surgical phase. The lowest PEEP level yielding the highest dynamic compliance value was identified and set as the optimal PEEP (Figure 1).

In both groups, SpO_2_ was targeted at 92–98% and end-tidal CO_2_ (EtCO_2_) at 35–45 mmHg, with FiO_2_ and respiratory rate adjusted accordingly on an individual basis. A mean arterial pressure of ≥65 mmHg was targeted throughout the intraoperative period; values falling below this threshold were defined as intraoperative hypotensive events and managed with appropriate doses of ephedrine or norepinephrine. Vasopressor doses and hypotensive episodes were recorded. At the end of the surgery, a standardized recruitment maneuver was applied to all patients at 40 cmH_2_O for 30 s. Arterial blood gas analyses were performed at predetermined time points: following induction, during pneumoperitoneum, and upon arrival at the postoperative care unit.

### 2.5. Postoperative Care Unit Period

Thirty minutes after arrival at the postoperative care unit, lung ultrasonography was performed by an experienced anesthesiologist who was blinded to group allocation, using a MyLab™ Seven ultrasound device (Esaote, Italy) equipped with a convex probe (2–5 MHz). Each hemithorax was divided into six anatomical regions, defined by three vertical lines drawn along the anterior axillary, parasternal, and posterior axillary lines, intersected by two imaginary horizontal lines positioned 1 cm above the nipple line and at the diaphragm level. Each region was systematically evaluated for the presence of lung sliding, A-lines, B-lines, consolidation, air bronchograms, pleural effusion, and pneumothorax. A total of 12 regions across both hemithoraces were scored in accordance with the modified lung ultrasound scoring system (mLUSS) described by Monastesse et al., and the resulting composite score was recorded for each patient [12].

In this scoring system, each of the 12 lung regions was assigned a score from 0 to 3 based on the degree of aeration loss. A score of 0 indicated normal aeration (0–2 B-lines); 1 indicated small loss of aeration (≥3 B-lines, or one or more small subpleural consolidations separated by a normal pleural line); 2 indicated moderate loss of aeration (multiple coalescent B-lines, or multiple small subpleural consolidations separated by a thickened or irregular pleural line); and 3 indicated severe loss of aeration (consolidation, or small subpleural consolidation exceeding 1 cm × 2 cm in diameter). Regional scores were summed to calculate the total mLUSS, yielding a score between 0 (no aeration loss) and 36 (maximum aeration loss); higher scores indicate greater pulmonary aeration loss and more severe atelectasis.

### 2.6. Postoperative Ward Period (Days 1, 2, and 3)

Patients fulfilling hemodynamic and respiratory stability criteria in the postoperative care unit were subsequently transferred to the ward. Until discharge, all patients were visited once daily and systematically assessed for oxygen requirements, peripheral oxygen saturation values, and the development of postoperative pulmonary complications. Postoperative pulmonary complications were defined as follows: pneumonia was diagnosed based on the presence of a new pulmonary infiltrate on chest radiography combined with at least two of the following criteria: fever (>38 °C), leukocytosis (>12,000/mm^3^), and purulent respiratory secretions. Atelectasis was defined as lobar or segmental collapse confirmed on chest radiography or computed tomography obtained on clinical indication. Pleural effusion was defined as a new fluid collection identified on imaging. Pneumothorax was defined as the presence of air in the pleural space, confirmed radiologically. Acute respiratory distress syndrome (ARDS) was defined according to the Berlin criteria. Respiratory failure was defined as a need for supplemental oxygen beyond postoperative day 2 or a requirement for non-invasive or invasive respiratory support after discharge from the postoperative care unit.

### 2.7. A Priori Power Analysis

The sample size of the study was calculated based on data derived from the study by Kim et al., in which a statistically significant difference in modified lung ultrasound scores was demonstrated between groups in patients who underwent laparoscopic surgery [13]. Power analysis performed using G*Power 3.1 software determined that a minimum of 28 patients per group was required to achieve a type I error rate (α) of 0.05 and a statistical power (1-β) of 0.95. Accounting for potential patient dropout and protocol deviations, the sample size was increased to 35 patients per group, yielding a total enrollment of 70. AI-assisted tools were used during manuscript preparation exclusively for linguistic editing purposes. No AI tools were used for data analysis, interpretation, or generation of scientific content.

### 2.8. Statistical Analysis

Statistical analyses were performed using SPSS (version 27.0, IBM Corp., Armonk, NY, USA) and GraphPad Prism (version 10.1.0 GraphPad Software, San Diego, CA, USA). The conformity of data to a normal distribution was evaluated using the Shapiro–Wilk test and Q-Q plots. Descriptive statistics are presented as numbers (n) and percentages (%) for categorical variables and as mean ± standard deviation, median, minimum, and maximum values for continuous variables.

### 2.9. Comparisons Between Groups

Comparisons of categorical variables (sex, type of surgery, ASA score, presence of OSAS, intraoperative hypotensive events, atelectasis, pneumonia, pneumothorax, pleural effusion, and ARDS) between the groups (CDYN and PEEP8) were performed using the chi-square test (*χ*^2^). In cases where the expected frequency was less than 5 (in 2 × 2 tables), Fisher’s exact test results were considered.

For the comparison of continuous variables (age, BMI, ICU length of stay, hospital length of stay, duration of surgery, perioperative vital parameters, blood gas values, ventilation parameters, fluid balance, vasopressor use, CRP values, and mLUSS scores) between the groups, an independent-samples *t*-test was used. Homogeneity of variances was assessed using Levene’s test, and in cases where the homogeneity assumption was violated, corrected *t*-test results (equal variances not assumed) were reported.

### 2.10. Within-Group Repeated Measurement Comparisons

The Friedman test (Related-Samples Two-Way Analysis of Variance by Ranks) was used to compare ventilation parameters (PPV, ETCO_2_, CDYN, PEEP, PLATO, PEAK, tidal volume, minute volume) at different time periods (intubation, ppt, ppt15, ppt30, ppt45, ppt60, ppt75) in both groups (CDYN and PEEP8). For parameters with significant differences, post hoc analysis with Bonferroni correction (pairwise comparisons) was applied to determine the time points at which the differences occurred.

The Friedman test was also used to evaluate temporal changes in perioperative blood gas parameters (pH, PaO_2_, PaCO_2_, SpO_2_, lactate, and P/F ratio) and postoperative CRP values, with Bonferroni-corrected post-hoc analysis performed for significant findings.

### 2.11. Significance Level

In all analyses, the statistical significance level was set at *p* < 0.05. The Bonferroni correction was applied for multiple comparisons to control for type I error, and adjusted significance values (adjusted *p*-values) were reported.

## 3. Results

**Figure 2:**
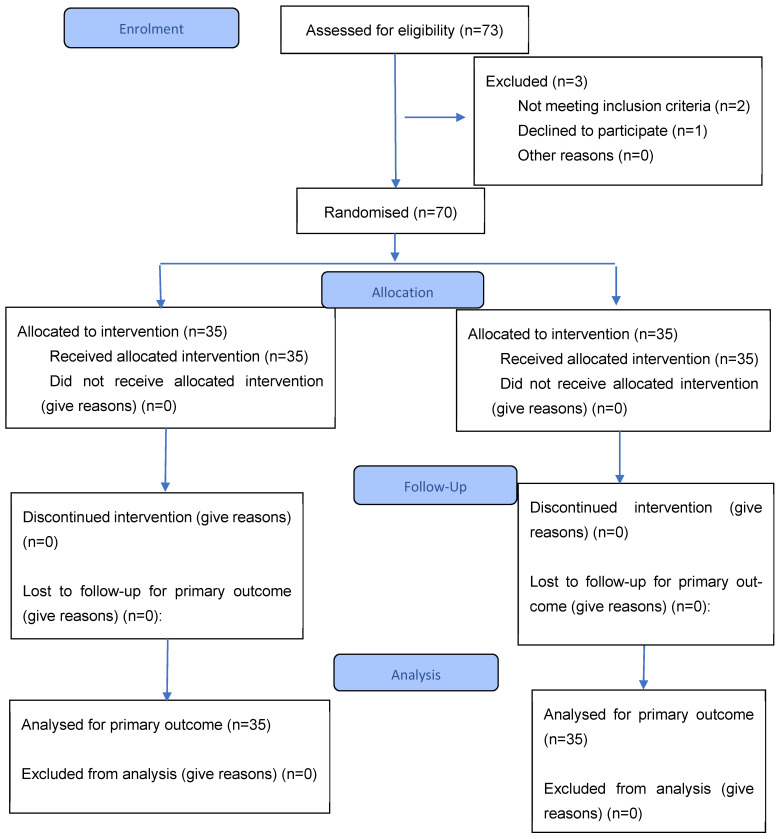
CONSORT diagram of patient recruitment.

A total of 73 patients were assessed for eligibility. Of these, 3 were excluded prior to enrollment: 2 did not meet the inclusion criteria, and 1 declined to participate (Figure 2). The remaining 70 patients provided written informed consent and were enrolled in the study, subsequently randomized in a 1:1 ratio to either the PEEP8 group (n = 35) or the CDYN group (n = 35). No patients were withdrawn from the study during data collection or statistical analysis; therefore, all randomized patients were included in the final analysis.

Baseline demographic and clinical characteristics of the study population are summarized in Table 1. The two groups were well-matched at baseline, with no statistically significant differences observed in sex distribution, age, body mass index (BMI), ASA physical status classification, surgical type, or prevalence of obstructive sleep apnea (all *p* > 0.05). Intraoperative variables, including pneumoperitoneum duration, total operative time, intraoperative blood loss, total volume of crystalloid fluid administered, urinary output, number of intraoperative hypotensive episodes, and total vasopressor consumption, were likewise comparable between the PEEP8 and CDYN groups (all *p* > 0.05).

The primary outcome of the study, the modified lung ultrasound score (mLUSS), was significantly lower in the CDYN group than in the PEEP8 group across all measured parameters, including the right hemithorax, left hemithorax, and total composite scores (all *p* < 0.001), indicating a markedly reduced degree of postoperative aeration loss in patients receiving individualized dynamic compliance-guided PEEP titration. These findings are summarized in Table 2.

With respect to postoperative clinical outcomes, the length of hospital stay was comparable between the CDYN and PEEP8 groups (2.97 ± 0.62 vs. 3.17 ± 0.57 days, *p* = 0.163). Intensive care unit admission was required in two patients in the CDYN group (5.7%) and in none of the patients in the PEEP8 group, with no statistically significant difference between the groups (*p* = 0.321). Regarding postoperative pulmonary complications, atelectasis was observed in 3 patients (4.3% of the total cohort), all of whom belonged to the PEEP8 group, with the difference approaching but not reaching statistical significance (χ^2^ = 3.134, *p* = 0.077). Pneumonia and pleural effusion were each observed in one patient (1.4%), whereas no cases of pneumothorax or acute respiratory distress syndrome (ARDS) were recorded in either group. The incidence of these complications did not differ significantly between the CDYN and PEEP8 groups (all *p* > 0.31). The overall incidence of postoperative pulmonary complications was low, occurring in three patients in the PEEP8 group (8.6%) and none in the CDYN group, although this difference did not reach statistical significance (*p* = 0.239).

Peripheral oxygen saturation (SpO_2_) was significantly higher in the CDYN group at all three postoperative assessment time points: on the day of surgery (PO0: 97.69 ± 1.83% vs. 96.34 ± 1.94%, *p* = 0.004), on postoperative day 1 (PO1: 97.97 ± 1.82% vs. 96.77 ± 1.91%, *p* = 0.009), and on postoperative day 2 (PO2: 97.89 ± 1.84% vs. 96.83 ± 1.62%, *p* = 0.013).

Inflammatory markers, as reflected by C-reactive protein (CRP) levels, did not differ significantly between the groups on either postoperative day 0 (16.04 ± 10.50 vs. 13.35 ± 7.36 mg/L, *p* = 0.219) or postoperative day 1 (55.06 ± 41.30 vs. 50.96 ± 50.69 mg/L, *p* = 0.712).

Arterial blood gas parameters, oxygenation indices, and lactate levels at the three intraoperative time points are summarized in Table 3.

At T1 (following induction), pH was significantly higher in the CDYN group (7.43 ± 0.04 vs. 7.40 ± 0.04, *p* = 0.006), and PaCO_2_ was significantly lower (36.60 ± 3.07 vs. 38.63 ± 4.30 mmHg, *p* = 0.026), while PaO_2_, SpO_2_, EtCO_2_, and P/F ratio did not differ significantly between groups (all *p* > 0.05).

At T2 (during pneumoperitoneum), no statistically significant differences were observed between the CDYN and PEEP8 groups in pH, PaO_2_, PaCO_2_, SpO_2_, P/F ratio, or lactate levels (all *p* > 0.05).

**Figure 3:**
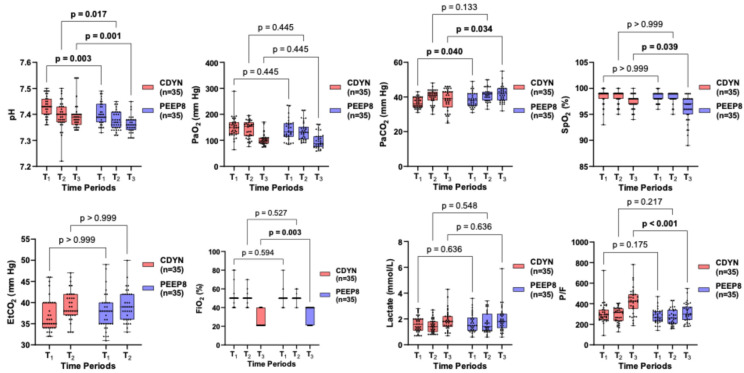
Perioperative arterial blood gas parameters, oxygenation indices, and lactate levels in the CDYN and PEEP8 groups at intraoperative time points. Friedman test. CDYN: dynamic compliance-guided PEEP group; PEEP8: fixed 8 cmH_2_O PEEP group; T_1_: post-induction; T_2_: during pneumoperitoneum; T_3_: postoperative care unit.

At T3 (postoperative care unit), the CDYN group demonstrated significantly more favorable respiratory parameters across multiple indices. The pH was significantly higher (7.40 ± 0.05 vs. 7.36 ± 0.03, *p* = 0.001); PaCO_2_ was significantly lower (38.37 ± 6.21 vs. 41.80 ± 4.70 mmHg, *p* = 0.011); and SpO_2_ was significantly higher (97.37 ± 1.37% vs. 96.14 ± 2.49%, *p* = 0.013) in the CDYN group. Notably, the P/F ratio was markedly higher in the CDYN group (425.11 ± 127.13 vs. 311.65 ± 92.59 mmHg, *p* < 0.001), reflecting a clinically meaningful improvement in oxygenation. Accordingly, the required supplemental nasal FiO_2_ at T3 was significantly lower in the CDYN group (25.89 ± 8.43% vs. 32.94 ± 9.31%, *p* = 0.001), indicating a reduced need for postoperative oxygen supplementation. PaO_2_ at T3 did not differ significantly between groups (*p* = 0.440). Lactate levels remained comparable between the two groups at all three time points (T1: *p* = 0.630; T2: *p* = 0.181; T3: *p* = 0.611) (Figure 3).

**Figure 4:**
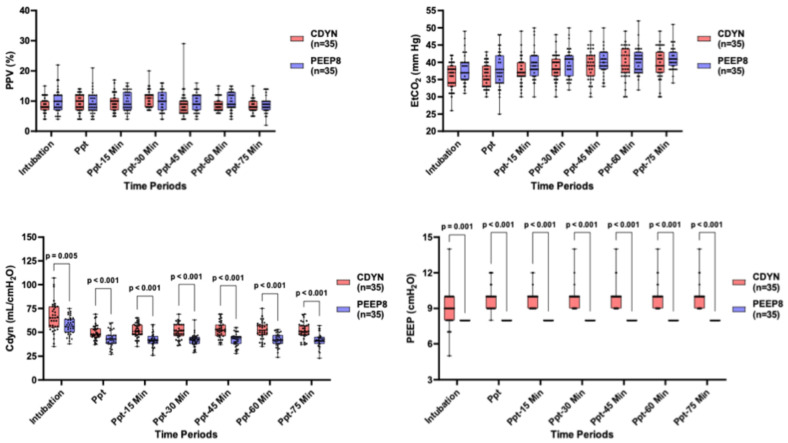
Intraoperative PPV, ETCO_2_, Cdyn, PEEP parameters in the CDYN and PEEP8 groups at sequential time points. Statistical comparisons between groups at each time point were performed using the Friedman test. *p* < 0.05 between groups. PPV: pulse pressure variation; EtCO_2_: end-tidal carbon dioxide; Cdyn: dynamic compliance; PEEP: positive end-expiratory pressure; Ppt: pneumoperitoneum; Min: minutes, CDYN: dynamic compliance-guided PEEP group; PEEP8: fixed 8 cmH_2_O PEEP group.

**Figure 5:**
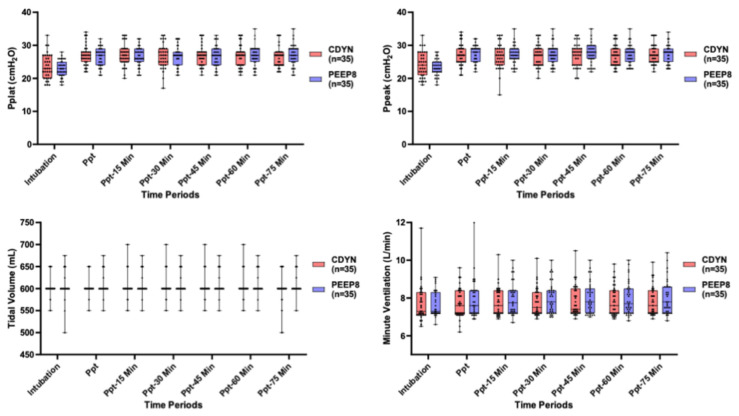
Intraoperative plato and peak airway pressure, tidal volume, and minute ventilation parameters in the CDYN and PEEP8 groups at sequential time points were performed using the Friedman test. *p* < 0.05 between groups. Pplat: plateau pressure; Ppeak: peak airway pressure; Ppt: pneumoperitoneum; Min: minutes; cmH_2_O: centimeters of water; mL: milliliters; L/min: liters per minute, CDYN: dynamic compliance-guided PEEP group; PEEP8: fixed 8 cmH_2_O PEEP group.

Throughout the intraoperative period, dynamic compliance and PEEP values were significantly higher in Group CDYN at all measurement time points (Figure 4). In contrast, no statistically significant differences were detected between the two groups in plateau pressure, peak airway pressure, tidal volume, minute ventilation, or pulse pressure variation at any intraoperative time point (Figure 5).

## 4. Discussion

In this prospective randomized controlled trial involving 70 patients with obesity undergoing laparoscopic bariatric surgery, we demonstrated that dynamic compliance-guided individualized PEEP titration (CDYN group) significantly reduced early postoperative pulmonary aeration loss, as assessed by the modified lung ultrasound score (mLUSS), compared to a fixed PEEP of 8 cmH_2_O (PEEP8 group). This difference was statistically significant in both hemithoraces.

The lower mLUSS scores in the CDYN group reflect ultrasound-detected aeration loss at an early postoperative time point rather than a definitive clinical diagnosis of atelectasis; however, early aeration loss is recognized as a risk factor for the subsequent development of postoperative pulmonary complications. Furthermore, the PaO_2_/FiO_2_ ratio was significantly higher in the CDYN group in the postoperative care unit, supporting the clinical relevance of this aeration benefit. The mLUSS scoring system, first described by Monastesse et al., is a validated and reproducible tool for objective quantitative assessment of atelectasis severity [12], and lung ultrasonography has been shown to have high sensitivity for detecting perioperative pulmonary complications [9,10]. The reliability of our results is further strengthened by the standardized application of this scoring system by a single experienced, blinded clinician, minimizing interobserver variability.

A study comparing fixed 8 cmH_2_O PEEP with dynamically titrated PEEP guided by compliance in patients scheduled for bariatric surgery reported less atelectasis in the dynamic compliance group on computed tomography imaging obtained 30 and 60 min following extubation [8]. However, PEEP titration in that study was performed only once after intubation using a decremental approach initiated from 25 cmH_2_O; no retitration was conducted during or after pneumoperitoneum. In another study conducted in patients undergoing laparoscopic bariatric surgery, fixed 10 cmH_2_O PEEP was compared with dynamically titrated PEEP guided by compliance; no significant difference was observed between the two groups with respect to the incidence of hypoxemia within the first 48 postoperative hours [14]. However, the intraoperative PaO_2_/FiO_2_ ratios were reported to be significantly higher in the group that received dynamic compliance-guided PEEP titration. In contrast to that study, no significant difference was observed in intraoperative PaO_2_/FiO_2_ ratios measured during pneumoperitoneum in our study. However, PaO_2_/FiO_2_ ratios in the postoperative care unit were significantly higher in the CDYN group. The relatively short pneumoperitoneum duration in our study may have been insufficient to allow a meaningful rise in arterial PaO_2_ during the intraoperative period. Intraoperative blood gas analyses revealed significantly higher pH values in the CDYN group both during pneumoperitoneum and in the postoperative measurements, suggesting that dynamic compliance-guided ventilation facilitates more effective CO_2_ elimination and better preservation of acid–base homeostasis. Consistent with these findings, Ghodraty et al. similarly demonstrated that optimized ventilation strategies improve gas exchange outcomes [1]. In our study, incremental PEEP titration was performed at three distinct time points: following intubation, at the initiation of pneumoperitoneum in the reverse Trendelenburg position, and upon return to the supine position after pneumoperitoneum deflation. At each titration point, the PEEP level yielding the highest dynamic compliance was identified, and patients were subsequently ventilated at that optimal value.

The physiological rationale for dynamic compliance-guided PEEP titration is well supported. In an experimental porcine model, Suarez-Sipmann et al. demonstrated a strong correlation between the PEEP level at which Cdyn reached its maximum value and the onset of lung collapse, establishing dynamic compliance as a reliable bedside tool for identifying the optimal PEEP required to prevent expiratory alveolar collapse [15]. Eichler et al. showed that esophageal pressure-guided titration in laparoscopic bariatric surgery required a mean PEEP of 16.7 cmH_2_O to achieve positive transpulmonary pressure, rising to 23.8 cmH_2_O after capnoperitoneum—confirming the profound influence of elevated intra-abdominal pressure on respiratory mechanics and the inadequacy of a single pre-incision titration [16]. A meta-analysis of PEEP strategies in patients with obesity undergoing laparoscopic surgery demonstrated that both high PEEP and individualized PEEP significantly improved PaO_2_/FiO_2_ ratios, dynamic compliance, and driving pressure compared to low PEEP [17]. In a secondary analysis by Simon et al., individualized PEEP titrated under EIT guidance yielded superior oxygenation and lower driving pressure compared to fixed PEEP strategies; the notably higher mean PEEP of 18 cmH_2_O in that study likely reflects the considerably higher BMI range of the study population (48–51 kg/m^2^) relative to our cohort [2].

In our study, dynamic compliance was significantly higher in the CDYN group at all intraoperative time points (*p* < 0.001), confirming that individualized titration improves pulmonary mechanics. Importantly, peak and plateau pressures did not differ significantly between groups (*p* > 0.437), indicating that the higher individualized PEEP values did not increase barotrauma risk—a finding consistent with those of Nestler et al., who similarly reported higher dynamic compliance with EIT-guided titration, though in contrast to our results, peak and plateau pressures were significantly elevated in their titrated group [18]. The comparable hemodynamic profiles between our groups—including heart rate, arterial blood pressure, lactate levels, and intraoperative hypotensive episodes—further confirm that dynamic compliance-guided PEEP titration did not compromise circulatory stability, likely reflecting the relatively moderate individualized PEEP values achieved in our cohort.

A randomized controlled study by Severac et al. conducted in patients undergoing bariatric surgery demonstrated that systematic alveolar recruitment maneuvers significantly reduced early postoperative pulmonary dysfunction [19]. However, this beneficial effect was confined to the early postoperative period, with no significant differences observed between groups in terms of pulmonary complication rates or length of hospital stay. An important interpretative consideration in our study is that the observed benefit in the CDYN group may partly reflect the repeated incremental titration maneuver itself rather than solely the resulting optimized PEEP level. At each of the three intraoperative time points, PEEP was incrementally increased in a stepwise manner, a process that inherently generates a recruitment-like effect on collapsed alveolar units. Since the control group did not undergo any analogous maneuver, the intervention in the CDYN group should be understood not simply as individualized PEEP optimization, but more precisely as repeated recruitment-like titration combined with individualized maintenance PEEP. This distinction is clinically meaningful and should be considered when interpreting the differences between the groups in aeration scores and oxygenation indices. This mechanism is broadly analogous to the planned recruitment maneuvers employed by Severac et al., and the overlap between these two effects cannot be fully disentangled in our study design.

Regarding postoperative outcomes, it is well established that intraoperative ventilation strategy alone does not determine postoperative pulmonary complication rates. Severac et al. demonstrated that systematic recruitment maneuvers reduced early postoperative pulmonary dysfunction but conferred no benefit on complication rates or hospital length of stay [19], and Nestler et al. similarly reported that intraoperative improvements with individualized PEEP did not translate into significant postoperative differences [18]. In a retrospective analysis of approximately 2000 patients with obesity, high PEEP with recruitment maneuvers offered no advantage over low PEEP in terms of pulmonary complications and was associated with higher hemodynamic adverse events [6]. A meta-analysis of one-lung ventilation studies demonstrated that compliance-guided individualized PEEP reduced postoperative pulmonary complication rates more effectively than driving pressure- or static compliance-based approaches [20], and EIT-based assessment in bariatric surgery confirmed that 10 cmH_2_O PEEP combined with repeated recruitment maneuvers prevented an increase in atelectasis at PACU discharge [21]. In our study, major complications were exceedingly rare in both groups; pneumonia and pleural effusion each occurred in a single patient (1.4%), with no ARDS or pneumothorax, and no significant between-group differences in any complication (*p* > 0.31).

In our study, subclinical aeration loss detected by lung ultrasonography was substantially more prevalent in the PEEP8 group, as reflected by significantly elevated mLUSS scores. During postoperative follow-up, the incidence of clinically defined atelectasis was 4.3% in the PEEP8 group, whereas no cases were observed in the CDYN group (*p* = 0.077). Based on these findings, it may be postulated that ultrasonographically detected atelectasis does not necessarily translate into clinically meaningful differences. However, larger-scale studies incorporating standardized postoperative care protocols, extended follow-up periods, and systematic screening are warranted to substantiate this hypothesis. The comparable rates of hospital length of stay, intensive care unit admission, and postoperative pulmonary complications between the groups suggest that the individualized PEEP strategy did not exert a significant influence on these outcomes. It should be acknowledged that the development of postoperative pulmonary complications is not solely governed by intraoperative ventilation strategy but is subject to the influence of numerous factors in the post-PACU period. Shallow breathing secondary to inadequate analgesia, failure to achieve early mobilization, insufficient respiratory exercises, and reduced preoperative pulmonary capacity are the principal contributors to atelectasis development. Critically, postoperative management—including analgesic regimen, supplemental oxygen delivery, mobilization timing, and respiratory physiotherapy—was not protocolized in the present study. Given that these factors can independently and substantially influence postoperative oxygenation and pulmonary complication rates, the absence of standardization represents a significant limitation that reduces the attributability of secondary postoperative outcomes to the intraoperative ventilation strategy alone. Future trials should incorporate a standardized postoperative care protocol to allow for a more rigorous assessment of the downstream clinical impact of individualized PEEP titration.

Postoperative SpO_2_ was consistently higher in the CDYN group on days 0, 1, and 2 (all *p* < 0.013), and supplemental nasal oxygen requirements were significantly lower (*p* = 0.001), indicating that patients in the PEEP8 group required more intensive respiratory support during the postoperative period. These differences in oxygenation support underscore the clinical relevance of the intraoperative aeration benefit achieved by individualized PEEP titration. As demonstrated by Ferrando et al., postoperative respiratory support strategies—including humidified high-flow nasal oxygen, which reduced postoperative hypoxemia from 80% to 28% and atelectasis from 77% to 31%—play a critical role in sustaining perioperative gains [22]. The integration of complementary strategies such as HFNO, prophylactic NIV, early mobilization, and respiratory physiotherapy is therefore essential to consolidate the intraoperative benefits observed in the CDYN group and to translate physiological improvements into clinically meaningful outcomes.

This study has several limitations. The single-center design limits generalizability, and validation across different surgical teams, anesthesia protocols, and patient populations is warranted. Outcomes were assessed only during the early postoperative period; long-term endpoints such as 30-day pulmonary complication rates and quality of life were not evaluated. Regarding lung ultrasonography, assessments were performed by a single experienced clinician without interobserver reliability testing, and preoperative baseline imaging was not obtained. Postoperative imaging beyond the PACU was performed only when clinically indicated. Although all patients with BMI > 35 kg/m^2^ were included, the sample size was insufficient for subgroup analyses stratified by obesity degree or phenotype. The relatively narrow PEEP range observed in the CDYN group (8.86–10.03 cmH_2_O) may limit the applicability of this approach in patients requiring higher PEEP levels. The use of a uniform recruitment maneuver (40 cmH_2_O for 30 s) precluded the evaluation of individualized recruitment strategies. An important interpretative limitation is that the observed benefits in the CDYN group cannot be attributed solely to individualized PEEP titration, as the repeated incremental titration procedure itself generates a recruitment effect. Since no analogous maneuver was applied in the control group, the intervention is more accurately described as repeated recruitment-like titration combined with individualized PEEP maintenance. Future trials should incorporate equivalent recruitment maneuvers in the control group to isolate the independent contribution of PEEP optimization. Finally, the absence of a standardized postoperative care protocol and the modest sample size may have limited statistical power to detect differences in postoperative pulmonary complication rates.

## 5. Conclusions

In conclusion, dynamic compliance-guided individualized PEEP titration, applied at three distinct intraoperative stages to account for the sequential physiological changes imposed by pneumoperitoneum and surgical positioning, represents a clinically feasible, safe, and lung-protective ventilation strategy that significantly reduces early postoperative pulmonary aeration loss in patients with obesity undergoing laparoscopic bariatric surgery. Reassessment of PEEP at critical intraoperative time points—guided by real-time dynamic compliance values displayed on modern anesthesia workstations—is of clinical importance in mitigating both alveolar overdistension and aeration loss without requiring additional equipment or complex calculations, thereby establishing it as a cost-effective and readily implementable approach to individualized perioperative lung protection.

Future studies should evaluate the efficacy of this approach across different degrees of obesity (BMI 35–40, 40–50, and >50 kg/m^2^) and obesity phenotypes (central vs. peripheral), as such investigations would yield valuable data for subgroup analyses. The absence of a standardized postoperative care protocol, including analgesic management, mobilization, and respiratory support, represents an additional limitation that may have influenced secondary outcome comparisons between groups. Future trials should incorporate a standardized postoperative care protocol to allow for a more rigorous assessment of the downstream clinical impact of individualized PEEP titration strategies. The combination of individualized recruitment maneuvers with dynamic compliance titration may further optimize the existing ventilation protocol, while real-time PEEP optimization through machine learning and artificial intelligence algorithms holds the potential to reduce clinician workload and deliver a more precise and automated approach to individualized ventilation management.

## Figures and Tables

**Table 1:** Baseline Demographic, clinical, and intraoperative characteristics of the study groups. BMI: body mass index; ASA: American Society of Anesthesiologists; OSAS: obstructive sleep apnea syndrome, CDYN: dynamic compliance-guided PEEP group; PEEP8: fixed 8 cmH_2_O PEEP group.

Parameters	Group CDYN (n = 35) (Mean ± SD)	Group PEEP8 (n = 35) (Mean ± SD)	*p*-Value
Sex			
Women n (%)	30 (%85.7)	29 (%82.9)	0.743 ^1^
Age (year)	41.83 ± 11.85	39.00 ± 12.33	0.331 ^1^
BMI (kg/m^2^)	44.34 ± 6.39	42.57 ± 4.61	0.187 ^1^
ASA score			
ASA II	25 (%71.4)	23 (%65.7)	0.607 ^1^
ASA III	10 (%28.6)	12 (%34.3)	
Pneumoperitoneum time (min)	83.86 ± 43.99	94.14 ± 60.08	0.417 ^1^
Total operative time (min)	104.71 ± 48.63	115.29 ± 60.77	0.424 ^1^
Surgical type			
Gastric bypass	5 (%14.3)	8 (%22.9)	0.357 ^1^
Sleeve gastrectomy	30 (%85.7)	27 (%77.1)	
OSAS prevalence	3 (%8.6)	5 (%14.3)	0.452 ^1^
Intraoperative blood loss (mL)	43.14 ± 36.20	66.29 ± 79.52	0.122 ^1^
Total volume of crystalloid (mL)	1511.43 ± 706.18	1734.29 ± 806.20	0.223 ^1^
Urine output (mL)	265.71 ± 212.75	319.71 ± 190.16	0.267 ^1^
Total vasopressor consumption (mg)	2.14 ± 4.42	1.14 ± 2.99	0.272 ^1^
Intraoperative hypotensive episode	8 (%22.9)	5 (%14.3)	0.357 ^1^

^1:^ chi-square test (*χ*^2^). Categorical variables are presented as numbers (percentages), continuous variables are presented as mean ± standard deviation.

**Table 2:** Lung ultrasound scores, oxygenation parameters, postoperative outcomes, and inflammatory markers.

Parameters	Group CDYN (Mean ± SD)	Group PEEP8 (Mean ± SD)	*p*-Value
mLUSS Right	1.26 ± 0.82	2.94 ± 1.14	<0.001 ^1^
mLUSS left	0.97 ± 0.71	2.89 ± 1.30	<0.001 ^1^
Total mLUSS	2.20 ± 1.16	5.80 ± 2.14	<0.001 ^1^
Hospital length of Stay (days)	2.97 ± 0.62	3.17 ± 0.57	0.163 ^1^
Intensive Care Unit Admission, n (%)	2 (%5.7)	0 (%0)	0.321 ^2^
PO complication, n (%)	0 (%0)	3 (%8.6)	0.239 ^2^
PO0SPO_2_ %	97.69 ± 1.83	96.34 ± 1.94	0.004 ^1^
PO1SPO_2_ %	97.97 ± 1.82	96.77 ± 1.91	0.009 ^1^
PO2SPO_2_ %	97.89 ± 1.84	96.83 ± 1.62	0.013 ^1^
PO0-CRP (mg/L)	16.04 ± 10.50	13.35 ± 7.36	0.219 ^1^
PO1-CRP (mg/L)	55.06 ± 41.30	50.96 ± 50.69	0.712 ^1^

Categorical variables are presented as numbers (percentages), continuous variables are presented as mean ± standard deviation. ^1^: independent-samples *t*-test; ^2^: Fisher’s exact test. mLUSS: modified lung ultrasound score; PO0-CRP: postoperative day 0 C-reactive protein level, PO1-CRP: postoperative day 1 C-reactive protein level, CDYN: dynamic compliance-guided PEEP group; PEEP8: fixed 8 cmH_2_O PEEP group.

**Table 3:** Perioperative arterial blood gas parameters, oxygenation indices, and lactate levels across intraoperative time points.

Parameters	Group CDYN (Mean ± SD)	Group PEEP8 (Mean ± SD)	*p*-Value
pH (T1)	7.43 ± 0.04	7.40 ± 0.04	0.006
PaO_2_ (T1) mmHg	149.83 ± 38.22	139.03 ± 39.00	0.246
PaCO_2_ (T1) mmHg	36.60 ± 3.07	38.63 ± 4.30	0.026
SpO_2_ (T1) %	98.40 ± 1.50	98.34 ± 0.97	0.850
EtCO_2_ (T1) mmHg	37.00 ± 3.98	37.63 ± 4.29	0.527
P/F (T1) mmHg	304.61 ± 98.13	276.72 ± 69.01	0.174
pH (T2)	7.39 ± 0.05	7.37 ± 0.03	0.051
PaO_2_ (T2) mmHg	141 ± 33	135 ± 34	0.42
PCO_2_ (T2) mmHg	39 ± 4	41 ± 3	0.132
SpO_2_ (T2) %	98.2 ±1.2	98.2 ± 1.0	1.00
Lactate (T2) mmol/L	1.44 ± 0.52	1.66 ± 0.79	0.181
P/F (T2)	288.73 ± 73.1	271.05 ± 75.0	0.322
pH (T3)	7.40 ± 0.05	7.36 ± 0.03	0.001
PaO_2_ (T3) mmHg	102.60 ± 22.10	97.83 ± 28.90	0.440
PCO_2_ (T3) mmHg	38.37 ± 6.21	41.80 ± 4.70	0.011
SpO_2_ (T3) %	97.37 ± 1.37	96.14 ± 2.49	0.013
Nasal FiO_2_ (T3) %	25.89 ± 8.43	32.94 ± 9.31	0.001
P/F (T3) mmHg	425.11 ± 127.13	311.65 ± 92.59	<0.001
Lactate (T1) mmol/L	1.61 ± 0.61	1.68 ± 0.72	0.630
Lactate (T2) mmol/L	1.44 ± 0.52	1.66 ± 0.79	0.181
Lactate (T3) mmol/L	1.89 ± 0.77	2.00 ± 0.99	0.611

Group comparisons were performed using an independent-samples *t*-test. Data are presented as mean ± standard deviation (SD). T1: following anesthesia induction; T2: during pneumoperitoneum; T3: postoperative care unit. P/F ratio: PaO_2_/FiO_2_ ratio; PaO_2_: arterial partial pressure of oxygen; PaCO_2_: arterial partial pressure of carbon dioxide; SpO_2_: peripheral oxygen saturation; EtCO_2_: end-tidal carbon dioxide; FiO_2_: fraction of inspired oxygen. CDYN: dynamic compliance-guided individualized PEEP group; PEEP8: fixed 8 cmH_2_O PEEP group.

## Data Availability

The data presented in this study are available upon reasonable request from the corresponding author.
